# Self-Care Practices and Knowledge of Hypertension Management Among Patients Attending Clinics in Tanzania: A Multi Centre Analytical Cross-sectional Study

**DOI:** 10.24248/eahrj.v9i2.849

**Published:** 2025-12-24

**Authors:** Julius Edward Ntwenya, Joseph Nyanda Shilole, Edmund Boniface Bunyaga, Elihuruma Eliufoo Stephano

**Affiliations:** a Department of Public Health and Community Nursing, School of Nursing and Public Health, The University of Dodoma, Dodoma, Tanzania; b Department of Clinical Nursing, School of Nursing and Public Health, The University of Dodoma, Dodoma, Tanzania; c Clinical Nursing Teaching and Research Section, The Second Xiangya Hospital of Central South University, Changsha, Hunan 410011, China

## Abstract

**Background:**

Hypertension is a serious public health problem. Adherence to the recommended self-care practices (SCPs) significantly prevented hypertension and abated its undesired related health outcomes. This study assessed the SCPs, knowledge of hypertension management, and factors associated with SCPs adherence among adults attending clinics in selected hospitals in Tanzania.

**Methods:**

A hospital-based analytical cross-sectional study was conducted among 311 adult hypertensive patients attending clinics in Dodoma and Dar-es-Salaam regions in Tanzania. This sample was systematically randomly selected during clinic visits, thus making a total of nine visits for the total sample. SCPs were assessed using the self-administered Hypertension Self-care Activity Level Effect Scale (H-SCALE). Analysis was done using SPSS, version 29.

**Results:**

The mean age of the surveyed hypertensive patients was 53.6 ± 7.5 years. Overall, 25.1% had good SCPs, 76.2% adhered to prescribed antihypertensive drugs, 99.0% were non-smokers, 95.2% were non-alcohol users, 59.5% observed recommended dietary plans, 64.3% had regular physical activities, and 66.2% observed a diverse set of recommended eating and lifestyle behaviour. 71.1% had adequate knowledge about hypertension management. On binary logistic regression analysis, high odds of SCP adherence among hypertensive patients was associated with the absence of a family history of hypertension (AOR=2.5, 95% CI=1.2–5.0, *P*=.01) and adequate knowledge (AOR=2.3, 95% CI=1.8–4.4, *P*=.003). Conversely, the absence of comorbidities was associated with lower odds of SCPs among hypertensive patients (AOR=0.5, CI=0.3–0.9, *P*=.03).

**Conclusion:**

This study found that most hypertensive patients had poor SCPs. There is a discrepancy between the level of knowledge and the day to day SCPs among hypertensive patients. Interventions should be responsive to how familial health history and comorbidities impact SCPs, thus empowering the patient with such knowledge and skills to manage hypertension properly.

## BACKGROUND

World Health Organization (WHO) defined hypertension as elevated blood pressure above 140/90 mmHg, a major global health problem affecting 33% of adults aged 30–79 years globally.^1^ It is expected that 1.5 billion people will be hypertensive by 2025, and 78% will live in lower and middle-income countries (LMICs).^2^ Hypertension is the largest and first contributor to deaths from non-communicable diseases (NCDs) worldwide, whereby its prevalence affects mostly the African region (36%).^1,2^

Hypertension is a growing health challenge in Tanzania, with the age-standardised prevalence ranges between 19% and 25%.^3,4^ Results of a nationwide survey showed that the prevalence of hypertension is currently at 25.9%.^5^ Geographically, the prevalence of hypertension varies across the regions from 19% in the rural up to 35% in urban areas, with the highest rates being observed amongst those aged 70 years and above at 70%.^6^ Additionally, a community-based survey conducted in rural Tanzania reported a hypertension prevalence of 29.3%.^7^

About 46% of the adults with high blood pressure are not aware, and only 42% of the people with high blood pressure are diagnosed with accompanied treatment.^8^ In contrast, it is widely recognised that hypertension is a killer disease and poses great health challenges. Self-care practices (SCPs) are, on the other hand, critical to managing blood pressure at the individual level. These practices focus on the ability of individuals, families, and communities to sustain health and prevent disease.^9^ SCPs encompass medication adherence, smoking cessation, maintaining a healthy body weight within the BMI range of 18.5 to 24.9, engaging in regular physical exercise, adhering to the Dietary Approaches to Stop Hypertension (DASH), adopting a low-salt and low-fat diet, and moderating alcohol consumption (men ≤ 2 and women ≤ 1 alcoholic drink).^2^ SCPs are intended to conserve health and well-being in patients’ interest by making certain day-to-day decisions and actions to control their illness.^10^

In general, adherence to recommended SCPs has long-term health benefits. For example, smoking cessation has direct as well as long-term benefits for patients with hypertension.^11^ Also, reducing dietary sodium intake to less than 2400 mg/day and implementing the DASH diet have a beneficial health effect, reducing blood pressure and its complications.^12^ Research showed those who did not add salt to the table had reduced odds of having uncontrolled BP by 40%.^13^ The DASH diet can reduce systolic blood pressure by 8 to 14 mmHg.^12^ Moreover, weight reduction significantly impacts blood pressure, whereby 1.6/1.1 mmHg is reduced for every kilogram lost.^14^ Minimal numbers of hypertensive adults aged 30 to 79 in the globe and Africa follow effective treatment guidelines at home and in the hospital.^1^

Sound knowledge of hypertension management among patients is a protective means against hypertension and contributes to improved prevention practices, treatment, and medication adherence.^15^ Studies show there is inadequate hypertension knowledge among patients, and most of the patients are unaware of SCPs.^16^ This led to an increase in morbidity and mortality due to increased blood pressure. Patients’ knowledge of hypertension management is a key factor in adherence to the recommended SCPs. However, there is limited multi-centre evidence from Tanzania on the specific levels of SCP adherence, knowledge, and the factors associated with them, which this study seeks to address. Therefore, this study describes the SCPs, knowledge of hypertension management, and socio-determinants among adults attending hypertension clinics in selected hospitals in Tanzania. The study findings are a cornerstone for the dissemination and advocacy of SCPs in preventing the burden of hypertension, especially in limited-resource settings.

## MATERIALS AND METHODS

### Study Design and Area

The study used a quantitative approach coupled with an analytical cross-sectional design. The study was conducted in two regions of referral hospitals within Tanzania in two different regions: Dodoma and Dar-es-Salaam. The data were collected from June to September 2020. Dar es Salaam region has 532 health facilities, including 436 dispensaries, 58 health centres, and 38 hospitals. Dodoma region has 469 health facilities, including 402 dispensaries, 41 health centres, and 26 hospitals. The region's dwellers aged 18 years and above are approximately 3.4 million and 1.6 million in Dar es Salaam and Dodoma, respectively.^17^

### Study Population, and Inclusion and Exclusion Criteria

The study involved hypertensive patients above 18 years, with at least 6 months of treatment, and recorded more than three clinic visits. All hypertensive patients who agreed and signed written informed consent participated in the study. Hypertensive patients in critical conditions that could impair responses, such as hypertensive patients with stroke, were not included in the study.

### Sample Size

The sample size for the quantitative study component was calculated based on the Kish-Leslie formula. We used a single population proportion formula assuming that the prevalence of SCP is 25.9% based on the Tanzania step survey (18), the standard normal variable, confidence level (α) (at 5% type 1 error, it is 1.96) and marginal error 5% (0.05). The calculation leads to a total sample of 295. A 10% non-response rate was added, and the adjusted sample size was 320. This study successfully collected data from 311 participants, a 97.2% response rate.

### Sampling Technique

The sampling strategy employed a two-stage approach, beginning with purposive sampling to select two strategically important regions (Dar es Salaam and Dodoma) and their respective regional referral hospitals (Amana and Dodoma Regional Referral Hospitals). This purposive selection ensured the representation of both Tanzania's commercial hub and administrative capital. The proportional allocation of participants between the two hospitals was determined based on patient attendance records from the preceding twelve months, ensuring appropriate distribution relative to each facility's patient volume. On designated data collection days, systematic random sampling was implemented to select eligible hypertensive patients as they arrived for their scheduled appointments, with every K-value^3^ meeting the inclusion criteria being invited to participate until the required sample size of 311 was achieved across both sites. The first patients in both hospitals were selected using the lottery method.

### Data Collection Tool and Procedures, and Variables Measurement

This study used a pretested and validated interviewer-administered questionnaire. SCPs were assessed on the Hypertension Self-Care Activity Level Effects (H-SCALE) standard tool with 31 questions about six recommended practices, including medication adherence, non-smoking, moderate or non-alcohol consumption, dietary plan, physical activities, and weight management (weight management was assessed through behaviours such as self-weighing, eating less to lose weight). The hypertensive patients who adhered to all six practices were regarded to have good SCPs in managing hypertension.

Medication adherence assessment involved asking three questions with a weekly reference to capture proper medication taking per week. Hypertensive patients who scored ≥21 days were regarded to have adhered to the recommended practice.^19^ Twelve questions assessed the dietary eating plan, asked several days a week, of which 9 were negatively phrased. The score ranged from 0–84. Those who scored less than 40 were considered to have adhered due to the negative phrasing of 9 items.

Assessment of weight management involved asking 10 questions on the Likert scale ranging from 10 to 50. Those who score ≥40 were considered to adhere to a recommendation. Regarding physical activity adherence, two questions were asked to capture the management of physical activity with a reference to one week with a total score range of 0 to 14; those who scored ≥8 were considered to have adhered to a recommendation.

Alcohol consumption was assessed by asking three questions adapted from the National Institute on Alcohol Abuse and Alcoholism (NIAAA).^20^ The score ranges from 0–21 days. The hypertensive patients with a score of 0 days were considered adhered. Lastly, non-smokers were asked one question about smoking tobacco, even just a single puff in a week; those who scored 0 days were considered to have adhered to a recommendation.

Patients’ knowledge level on hypertension management was assessed by 11 questions with dichotomous correct or incorrect adapted from another study.^21^ The hypertensive patients who scored more than 9 questions were regarded to have adequate knowledge.

Data collection was performed by four different trained research assistants who were qualified nurses with bachelor's degrees. We collected data during working time in the internal medicine clinics. The survey instrument was developed in English and then translated into Swahili languages to better understand and train research assistants. The translation used back-and-forth translation to ensure correctness and consistency. The data collection tool and procedures were piloted on 25 study participants. The data obtained from the pretesting were not used for the final analysis. The results were used to check the clarity, simplicity, understandability, consistency, and coherence of the questionnaire of the study tool. Strong supervision by the supervisor as assigned was maintained throughout the pretesting and final data collection phase.

### Data Analysis

Data were analysed using SPSS version 29. Descriptive analyses used frequency, percent, mean, and standard deviation. The categorical variables were tested using the Chi-square test, and a *P* value of less than .05 was considered statistically significant. Bivariate and binary logistic regression was done to test the association between the independent variables (age groups, family history, comorbidity, years with hypertension, occupation, and knowledge) and the outcome variable (SCPs on managing hypertension). The variables with a p-value of <0.25 from the univariate model were pre-determined to be added to the multivariable model to find the associated factors. The factors were presented in the adjusted odd ratio (AOR) with a 95% confidence interval.

### Ethical Considerations

The study's ethical clearance was obtained from the Institutional Research Review Committee (IRREC) and the President's Office, Regional Administration, and Local Government Authority (PORALG). Before participation, permission in hospitals was obtained from the medical in-charge. The participants were provided with detailed information about the research objectives, potential benefits, guarantees of confidentiality, the lack of any sort of risks, as well as the right to withdraw at any stage. Each respondent had provided the consent verbally and written before the data collection process. The study upheld confidentiality standards by ensuring anonymous data collection at all stages.

## RESULTS

### Demographic and Socio-Economic Characteristics of Hypertensive Patients

Of 311 interviewed hypertensive patients, 64.3% belonged to the 40–59 age group, and the mean age was 53.6±7.5 years. Females were 58.8%, of whom 71.7% were married, 53.4% had primary education, and 1% had no opportunity for formal education training, 53.1% had health insurance coverage, 11.9% lived alone, and 46.3% had an average monthly income of less than 270,000 Tsh (equivalent to 116.88 US$). Results show that about 47.9% of surveyed hypertensive patients had other comorbidities; of these, 7.7% had multiple comorbidities. The identified comorbidities were diabetes, asthma, retinopathy, heart failure, kidney disorders, neuropathy, and liver diseases with a prevalence of 21.5%, 10.0%, 3.5%, 1.9%, 1.3%, 1.3 and 1.0%, respectively. (Table 1).

**TABLE 1:** Profile of Studied Hypertensive Patients

Variables	n	%
Age group (years)		
18–39	35	11.3
40–59	200	64.3
60+	76	24.4
Sex		
Female	181	58.8
Male	130	41.2
Level of education		
Primary	166	53.4
Secondary	88	28.3
College	54	17.4
Non-formal	3	1.0
Participant income per month		
<270,000Tsh	144	46.3
270,001–520,000Tsh	81	26.0
520,001–760,000Tsh	70	22.5
760001–1000000Tsh	2	0.6
>1, 000,001Tsh	14	4.5
Health insurance coverage		
Yes	146	46.9
No	165	53.1
Live alone		
Yes	37	11.9
No	274	88.1

### Prevalence of Self-Care Practices Among Studied Hypertensive Patients

Overall, 25.1% of the hypertensive patients attending clinics in the selected hospitals had good SCPs. Patients adherence to medication, dietary plan, recommended weight management behaviours and physical activities is depicted in Table 2.

**TABLE 2:** Hypertensive Patients’ Adherence to Self-Care Practices (N=311)

Variables	n	%
Medication adherence		
Adherence	237	76.2
Non-adherence	74	23.8
Dietary plan		
Adherence	185	59.5
Non-adherence	126	40.5
Physical activity		
Adherence	200	35.7
Non-adherence	111	64.3
Non-smoking		
Adherence	308	99.0
Non-adherence	3	1.0
Weight management status		
Adherence	206	66.2
Non-adherence	105	33.8
Alcohol consumption		
Adherence	296	95.2
Non-adherence	15	4.8

### Patients’ Knowledge on Hypertension Management

The study found that only 71.1% of the hypertensive patients attending clinics in selected hospitals in Tanzania had adequate knowledge of hypertension management. The analysis of knowledge domains revealed four major knowledge patterns. The knowledge patterns identified were named based on the knowledge components with higher factor loading scores: I. Measurement of blood pressure, II. Consumption of a diet of less salt, III. Physical activity, and lastly, IV. The seriousness of hypertension. The patients were more knowledgeable on blood pressure measurement units, such as recognising the 140/80 mmHg blood pressure value. Other items for which a majority were knowledgeable include a diet of less salt, weight management, and planned physical activity, which were less knowledgeable by the patients (Table 3).

**TABLE 3:** Hypertension Management Knowledge Pattern

Domains	Knowledge pattern on self-care practices			
	Measurement of BP	Physical activity	A diet of less salts	Seriousness of hypertension
A blood pressure level of more than 140/80 is considered to be high	0.877	–	−0.325	–
An individual with hypertension should go for check-ups regularly	0.829	−0.332	0.294	–
A diet which contains fruits and vegetables is good for a person with Hypertension	0.774	0.415	–	–
Once persons develop high blood pressure, they become a lifetime consumer of medicine	0.660	−0.593	–	–
Smoking cigarettes has no negative effect on persons with Hypertension	0.573	0.566	–	–
Increasing physical exercise decreases the blood pressure of a person with hypertension.	0.369	0.849	–	–
Drinking alcohol has a negative effect on persons with hypertension.	0.636	−0.650	–	–
A diet consisting of less salt is good for a person with hypertension	–	0.323	0.837	–
Losing weight can help to lower blood pressure	0.427	–	0.816	–
Hypertension can cause stroke	0.326	0.421	−0.625	–
Hypertension is a serious condition that can lead to complications and often has no warning signs and symptoms.	–	–	–	0.986

### Factors Associated With Self-Care Practices

Binary logistic regression analysis revealed that absence of a family history of hypertension was significantly associated with higher odds of SCPs among hypertensive patients attending the clinic (AOR=2.5, 95% CI = 1.2–5.0). Also, Hypertensive patients with adequate knowledge demonstrated higher odds of engaging in SCPs (AOR=2.3, 95% CI = 1.9–4.4). On the other hand, our finding revealed another factor: the absence of comorbidities was associated with lower odds of SCPs among hypertensive patients (AOR=0.5, CI=0.3–0.9,) (Table 4).

**TABLE 4:** Binary Logistic Regression of SCPs, Patients’ Profile and Knowledge on Hypertension Management

Variable	95% CI			*p*-*value*	95% CI			*p*-*value*
	COR	Lower	Upper		AOR	Lower	Upper	
Age group (years)								
18–39	0.3	0.1	1.1	.1	0.7	0.1	3.5	.65
40–59	1.3	0.7	2.4	.4	1.5	0.6	3.8	.40
60+	1							
Family history								
No	2.7	1.5	4.5	.001	2.5	1.2	5.0	.01
Reference	1							
Comorbidity								
No	2.1	1.2	3.6	.001	0.5	0.3	0.9	.03
Yes (ref)	1							
Years with hypertension								
<4 years (ref)	1							
>5 years	2.7	1.2	6.3	.01	0.6	0.2	1.7	.34
Occupation								
Peasants	2.6	1.4	4.9	.02	0.5	0.2	1.2	.13
Employed (ref)	1							
Knowledge								
Reference	1							
Adequate	2.2	1.2	3.7	.01	2.3	1.8	4.4	.03

AOR= Adjusted Odd Ratio; COR= Crude Odd Ratio; CI= Confidence Interval

## DISCUSSION

In this study, adherence to recommended SCPs for the management of hypertension among hypertensive patients was found to be low. The adherence to SCPs is low in the sense that the majority of hypertensive patients did not meet the recommended practices, such as regular physical activity, low salt diet intake, weight management, proper medication intake, moderate alcohol intake, and non-smoking. The study finding is consistent with the studies done in Nigeria^22^ and Ethiopia,^2^ where there is low adherence to recommended hypertension management practices among hypertensive patients attending clinics in some places. Another study done in Ethiopia reported that more than half of their respondents had overall poor SCP.^23^

Significantly, the study found high SCP on dietary plans compared to those done in China and Ethiopia.^10,24^ On the other hand, the study conducted in Iran showed that a low-salt diet was less adherent.^25^ The observed difference between the studies could be due to variations in culture, beliefs, and food preparation and intake knowledge. The health intervention should be based on the availability and affordability of food and the culture of people in the food process and preparation.

Our study found that a good proportion of the hypertensive clients attending clinics at the selected study sites were non-adherent to the recommended medication as per the physician's prescriptions. Medication adherence-related challenges have also been reported in similar studies conducted in Tanzania, South India, and Palestine.^26–28^ This can, therefore, be an avenue for making the adult population conscious of adherence to their antihypertensive drugs to prevent further complications of hypertension. These thus provide an avenue for collaboration between the patient and healthcare provider.

Several factors were associated with SCPs, one of them is the absence of a family history of hypertension. The strong relationship between the absence of a family history of hypertension and the higher odds of doing SCPs in hypertensive patients in the clinic indicates the potential influence of family health background on health behaviours. Those not having a family history of hypertension would perceive they were at a lesser risk of having the disease and would be extra careful in taking preventive measures and practices of self-care. The same findings were found in the study conducted in Dessie, Ethiopia^29^ and another study in Nepal.^30^ These only underlines that the consideration of the familial health history is to be regarded in the course of the management of hypertension, as it may be a good indication of this or that the individual needs some targeted interventions and education about the importance of lifestyle changes and regular checking to reduce the risk of hypertension or its complications.

Another factor highlights the good role of health literacy in managing hypertension, where there is an association between hypertensive patients with sufficient knowledge and increased odds of SCP. People well aware of hypertension, its risk factors, and SCPs, on the other hand, are much better prepared to make choices regarding their health. This was supported by another study that shows the elaboration of educational programs for the patients in the improvement of knowledge and awareness, the recognition of hypertension, and the various strategies of control, which are important determinants.^31^ Also, another study showed that empowering patients with relevant knowledge and skills to manage the disease adequately. Healthcare professionals enable them selfefficacy to engage in proactive behaviours such as medication adherence, modification of diet, exercise regularly, and monitoring blood pressure.^32^ Educational interventions tailored to filling the gap in knowledge of the individual patient and encouraging SCPs may also contribute to improving the health status and quality of life with fewer complications due to hypertension among those affected.

Also, another factor was the absence of comorbidity. Our findings showed the reduced likelihood of engaging in SCPs amongst hypertensive patients without comorbidities. Paradoxically, this points to the fact that even though SCPs seem to be poor in those without comorbidities, it hints at special challenges for hypertensive patients with comorbidities.^33^ Besides, people with comorbidities may compete for the same attention and resources. Besides, an integrated care model that caters to the patient's holistic needs with comorbidity can enhance better coordination of the health care service.^34^ The notable sign flips for between the crude (COR=2.1) and adjusted (AOR=0.5) models is a significant finding, indicating substantial confounding. This reversal suggests that, after controlling for other influential factors, individuals with comorbidities are actually more likely to engage in good SCPs.

This study is limited by only involving hypertensive patients attending regional referral hospital clinics. The study could have missed important information from hypertensive patients who could not attend hypertension clinics and those who attended private hospitals. Also, recall bias could affect the study findings.

## CONCLUSIONS

In conclusion, the present study findings clarify the dire need for interventional efforts to improve adherence to SCPs among hypertensive patients attending clinics. While medication adherence was relatively high, adherence to other recommended self-care behaviours was low, This make health education and awareness programs that are attuned to the needs of hypertensive individuals highly crucial. Such worldwide efforts should advance literacy in health, and cultural and contextual interventions should be able to influence self-care behaviours and facilitate synergistic efforts for the patient and the healthcare providers. More so, interventions should be responsive to how familial health history and comorbidities impact SCPs, thus empowering the patient with such knowledge and skills to manage hypertension properly. This will be realised by educating and extending support to the integrated care models in developing these healthcare systems that will reduce the increased burden of hypertension and its complications to the people of the world.
